# Longitudinal Assessment of Mental Health Sequelae in COVID-19 Survivors: A Cohort Study

**DOI:** 10.7759/cureus.91010

**Published:** 2025-08-26

**Authors:** Garima Misra, Vandana Valluri

**Affiliations:** 1 Community Medicine, Employees' State Insurance Corporation (ESIC) Medical College and Hospital, Hyderabad, IND; 2 Community Medicine, Andhra Medical College, Vishakhapatnam, IND

**Keywords:** anxiety outcomes, covid-19 survivors, longitudinal study, mental health recovery, post-acute sequelae (pasc)

## Abstract

Introduction: The long-term consequences of COVID-19, also known as post-acute sequelae of SARS-CoV-2 infection (PASC), remain incompletely understood. Early studies have highlighted persistent multi-system symptoms, including anxiety, but data beyond one year are limited. This study aimed to evaluate the incidence of anxiety symptoms over three years among COVID-19 survivors. This cohort study aimed to investigate the longitudinal outcomes of COVID-19 survivors, with a focus on the persistence and development of sequelae.

Methods: The study enrolled a cohort of 109 subjects who were admitted with real-time polymerase chain reaction (RT-PCR)-confirmed COVID-19 in a tertiary care hospital in south-central India. Due to the retrospective enrollment in 2023, participants were interviewed to recall their anxiety status at discharge (2021) and one year after discharge (2022). This was followed by a prospective assessment of anxiety, two years (2023), and three years (2024) after discharge. The study involved the collection of quantitative data using a standardized questionnaire based on the Hamilton Anxiety Rating Scale (HAM-A), sent to the subjects via non-contact methods such as digital platforms or telephonic conversations. After data gathering, the data were tabulated on a Microsoft Excel spreadsheet and analyzed with the help of the IBM SPSS trial version 21. Statistical analysis included descriptive statistics, chi-square tests, and ordinal logistic regression.

Results: The mean age of the study subjects was 36.08 ± 15.31 years, with the majority being females. Corresponding to the time of discharge from the hospital, varying levels of anxiety were found among the participants, with most of them having mild anxiety, followed by severe anxiety and mild to moderate anxiety. The major contributors to the severe as well as mild to moderate anxiety were impaired intellectual abilities, insomnia, and depressed mood. The mean baseline HAM-A score at the time of discharge, one year, two years, and three years after discharge were 17.06 ± 10.71, 10.46 ± 8.38, 4.32 ± 5.21, and 1.13 ± 2.58, respectively.

Conclusion: Longitudinal follow-up showed a notable improvement in anxiety scores over a period of three years, with the mean score falling drastically at each year, suggesting a positive trend in mental health recovery. Findings support the need for integrated long-term mental health support and the need for primary case mental health screening.

## Introduction

The COVID-19 pandemic, caused by the emergence of the SARS-CoV-2 virus in late 2019, profoundly transformed global healthcare systems and economies [1-3]. It led to a worldwide economic crisis, especially affecting the low and middle-income countries [4]. While much is known about the immediate effects of acute COVID-19 infection, it has become increasingly clear that many survivors experience lingering health problems long after the initial illness. This condition, often referred to as "long COVID" or "post-acute sequelae of SARS-CoV-2 infection" (PASC), has emerged as a major concern for public health worldwide [5].

Studies have shown that the impact of COVID-19 extends well beyond the acute infection phase, with survivors reporting a wide array of symptoms that affect various organ systems [6,7]. These symptoms include neurological issues such as fatigue, brain fog, and confusion; cardiovascular complications like inflammation-induced arrhythmias; and persistent respiratory problems, including shortness of breath and increased risk of blood clots in the lungs. Patients have reported other chronic symptoms such as tinnitus, joint pain, and depression, highlighting the complex and multifaceted nature of long COVID [5].

The psychological toll of the pandemic has been significant as well. Beyond the fear of contracting the virus, many individuals have suffered from feelings of isolation, loss of freedom, and uncertainty about their health futures [6]. The strict safety protocols implemented to curb virus spread have inadvertently contributed to a rise in mental health challenges, including increased substance abuse and higher suicide rates. These mental health effects have compounded the difficulties faced by COVID-19 survivors, creating a broader crisis that affects communities and healthcare systems globally [6].

Early research estimated that between 10% and 30% of COVID-19 patients continue to experience symptoms for more than 12 weeks, regardless of how severe their initial infection was [5]. However, most studies have only followed patients for up to a year, leaving many questions about the long-term trajectory of these symptoms unanswered. Scientists are investigating several possible biological mechanisms behind long-term COVID-19, including persistent inflammation, autoimmune reactions, viral remnants in tissues, and damage to blood vessel linings. The diversity of symptoms suggests that multiple underlying processes may contribute simultaneously [5].

Beyond health, the pandemic has had far-reaching effects on employment, education, food security, small businesses, and social interactions, all of which have further strained mental well-being [1-3]. Despite the growing demand for mental health services, many regions have struggled to provide adequate support. Unlike other illnesses, COVID-19 has generated persistent feelings of fear, loneliness, irritability, and alienation even after recovery. Some individuals have developed obsessive-compulsive behaviors related to hygiene and respiratory precautions, underscoring the pandemic’s profound psychological impact [1-7].

The present study was an attempt to gather evidence on the long-term mental health effects of COVID-19, with an ultimate aim to aid in the rehabilitation of the survivors and bring their quality of life to near normalcy.

Objectives

The primary objective of this study was to understand the incidence of anxiety among COVID-19 survivors and to highlight the psychological consequences of the unprecedented pandemic. The primary outcome of the study was the change in the anxiety levels (Hamilton Anxiety Rating Scale (HAM-A) scores) among the COVID-19 survivors over a period of three years after COVID-19. 

## Materials and methods

This cohort study was conducted at a tertiary teaching hospital in south-central India to evaluate the longitudinal mental health outcomes among COVID-19 survivors admitted during one week of high hospitalizations (September 21-28, 2021). Ethical approval was obtained from the Institutional Ethics Committee (IEC number ESICMC/SNR/IEC-F523/03-2023), and oral informed consent was obtained from all participants after explaining the study purpose and confidentiality measures. Based on McGinty et al.’s reported 13.6% prevalence of psychological distress and using the standard sample size formula with a 7% margin of error, 92 participants were required; to account for attrition, 109 COVID-19 patients aged 18-60 years with real-time polymerase chain reaction (RT-PCR) confirmation (inclusion criteria) and no pre-existing psychiatric or chronic illnesses, pregnancy, or lactation were consecutively enrolled (exclusion criteria) [8].

Anxiety symptoms were assessed using the 14-item HAM-A, which rated severity from 0 (absent), 1 (mild), 2 (moderate), 3 (severe) to 4 (very severe), administered in English, Telugu, or Hindi via telephone or secure digital platforms according to participant preference (see Appendix 1) [9,10]. Due to retrospective enrollment in 2023, participants were interviewed to recall their anxiety status at discharge (September 2021) and one year after discharge (2022) in two separate interview sessions conducted in March 2023 and April 2023. This was followed by prospective assessments at two and three years after discharge (September 2023 and 2024, respectively), conducted using the same validated instrument. Data were anonymized and coded to protect confidentiality before entry into Microsoft Excel and were analyzed with IBM SPSS version 21; ordinal data were reported as medians with interquartile ranges, continuous data as means with SDs, and categorical data as frequencies and percentages, with statistical significance set at p < 0.05. 

## Results

The present study was conducted to understand the incidence of anxiety among COVID-19 survivors and to highlight the psychological consequences of the unprecedented pandemic. The primary outcome of the study was the change in the anxiety levels (HAM-A scores) among the COVID-19 survivors across four different time points: at discharge (2021, assessed retrospectively), one year post discharge (2022, assessed retrospectively), two years post discharge (2023, assessed prospectively), and three years post discharge (2024, assessed prospectively). 

**Figure 1:**
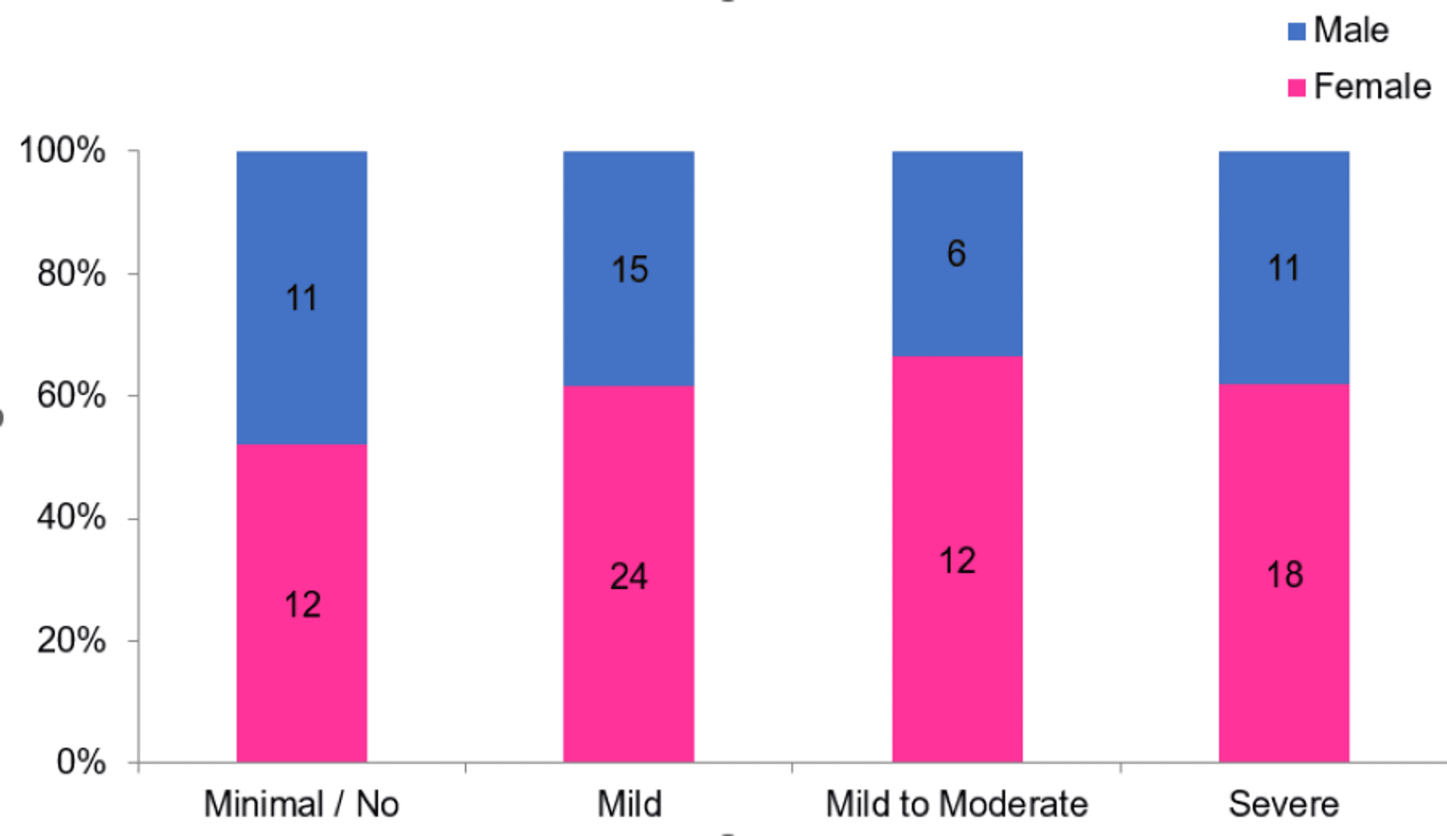
The component bar diagram for gender-wise distribution of study participants in each anxiety category

The sociodemographic characteristics of the study participants are given in Table 1 and Figure 1.

**Table 1:** Sociodemographic characteristics of the study participants

	Number (N)	Percent (%)
Age		
17-25	50	46
26-34	7	6
35-43	7	6
44-52	34	31
53-61	5	5
62-70	4	4
71-79	2	2
Gender		
Male	43	39
Female	66	61

The mean age of the study participants was 36.1 ± 15.31 years. The majority of the participants were female, 66 (60.6%), as compared to 43 (39.4%) who were male. The mean baseline HAM-A score at the time of discharge in 2021 (assessed in the first interview in March 2023 retrospectively) was 17.1 ± 10.71. The majority of the participants had mild anxiety (39 (35.8%)), followed by severe anxiety (29 (26.6%)) and mild to moderate anxiety (18 (16.5%)). However, 23 (21.1%) participants had minimal or no anxiety. Among participants with severe anxiety, 9 (31.0%) reported problems with concentration, memory, and decision-making, 8 (27.6%) had insomnia, and 8 (27.6%) experienced depressed mood. Although women comprised more than 60% of all three categories of anxiety, their predominance was highest among the participants with mild to moderate anxiety (12 (66.7%)). However, this association was not found to be statistically significant.

The second interview, conducted in April 2023 to assess the anxiety of the participants one year after discharge (in 2022), retrospectively showed that although majority (44 (40.4%)) of the participants had minimal or no anxiety, 24 (22%) participants each had mild and mild to moderate anxiety and 3 (2.8%) participants had severe anxiety at that time, whereas 14 (12.8%) were lost to follow-up. The mean HAM-A score was drastically reduced to 10.5 ± 8.38. 

The third interview, conducted in September 2023 to assess the anxiety of the participants two years after discharge (in 2023), prospectively showed further improvements with only 1 (0.9%) participant having severe anxiety, 2 (1.8%) having mild to moderate anxiety, and 22 (20.2%) having mild anxiety, while 66 (60.6%) showed minimal or no anxiety. The mean HAM-A score was 4.32 ± 5.21. By this time, 18 (16.5%) were lost to follow-up.

The fourth interview, conducted in September 2024 to assess the anxiety of the participants three years after discharge (in 2024), prospectively gave a mean HAM-A score of merely 1.13 ± 2.58 and showed that 6 (5.5%) participants continued to have mild anxiety, while 84 (77.1%) had minimal or no anxiety, and 19 (17.4%) were lost to follow-up. 

Altogether, the study that recruited 109 participants showed a loss to follow-up from 19 (17.4%) patients. Hence, the final sample size at the end of the study was 90. 

**Figure 2:**
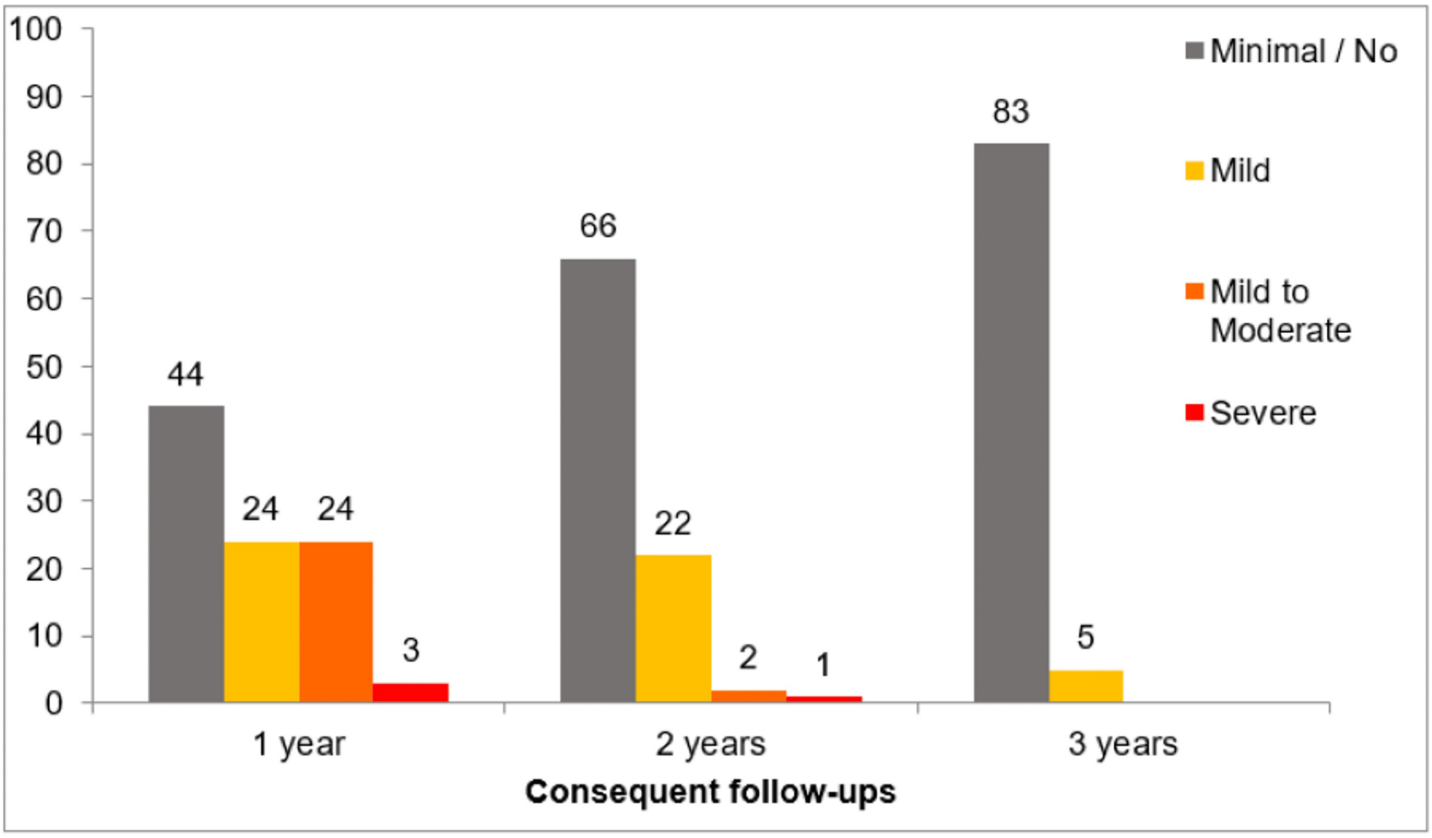
Anxiety profile of the study participants The consequent follow-ups here refer to the interviews conducted in April 2023, September 2023, and September 2024 to assess the anxiety profiles of the participants one, two, and three years post discharge, respectively.

The distribution of the study participants based on their anxiety categories, corresponding to their anxiety profiles they had in 2022 (one year after discharge), 2023 (two years after discharge), and 2024 (three years after discharge), are represented in Table 2 and Figure 2.

**Table 2:** Distribution of anxiety categories of the study participants at discharge and one, two, and three years post discharge Anxiety category at discharge (2021): Assessed retrospectively in March 2023 Anxiety category one year after discharge (2022): Assessed retrospectively in April 2023 Anxiety category two years after discharge (2023): Assessed prospectively in September 2023 Anxiety category three years after discharge (2024): Assessed prospectively in September 2024

Anxiety categories	At discharge		1 year after discharge		2 years after discharge		3 years after discharge	
	N	%	N	%	N	%	N	%
Minimal/No	23	21	44	40	66	61	84	77
Mild	39	36	24	22	22	20	6	6
Mild to Moderate	18	17	24	22	2	2	0	0
Severe	29	27	3	3	1	1	0	0
Lost to Follow-up	0	0	14	13	18	17	19	17

A one-way repeated measures analysis of variance (ANOVA) revealed a significant difference among the mean total HAM-A scores across four assessment points (at discharge, one year, two years, three years, and four years post discharge), with F = 78.212, p < 0.001, leading to the rejection of the null hypothesis and confirming statistically significant differences in the mean total score at these time points. The effect size (η²) was 0.42, indicating that 42% of the variance in the dependent variable is attributable to the time effect, which reflects a large and meaningful impact in the anxiety scores. Meanwhile, the mean HAM-A scores at the selected time points did not differ significantly between the genders and there was no correlation between age and the total HAM-A scores.

The mean HAM-A scores corresponding to different points of time are represented in Table 3, and the repeated ANOVA to test the significance of the differences between the mean HAM-A scores corresponding to different points of time are represented in Table 4 as follows. 

**Table 3:** Mean HAM-A scores corresponding to different points of time HAM-A scores at discharge (2021): Assessed retrospectively in March 2023 HAM-A scores one year after discharge (2022): Assessed retrospectively in April 2023 HAM-A scores two years after discharge (2023): Assessed prospectively in September 2023 HAM-A scores three years after discharge (2024): Assessed prospectively in September 2024 HAM-A: Hamilton Anxiety Rating Scale

HAM-A scores	Mean ± SD
At discharge	17.05 ± 10.70
One year after discharge	14 ± 12.11
Two years after discharge	8.40 ± 10.36
Three years after discharge	3.72 ± 6.13

**Table 4:** Repeated ANOVA to test the significance of the differences between the mean HAM-A scores corresponding to different points of time df: Degrees of freedom; F: F-ratio; p: p value; ANOVA: Analysis of variance; HAM-A: Hamilton Anxiety Rating Scale

	Type III sum of squares	df	Mean square	F	p	η2
Time	11463.18	3	3821.06	78.212	<0.001	0.42
Error	15829.06	324	48.85			

## Discussion

COVID-19 has had profound effects on global health due to the plethora of physical health implications and scarring psychological consequences. The present study was aimed at exploring the long-term psychological effects of COVID-19 over a period of three years among 109 COVID-19 survivors. The HAM-A was utilized to assess the anxiety among the subjects corresponding to the time of their discharge from the hospital, and later until three years after discharge. This scale was used to assess both the psychological and somatic symptoms of anxiety. It was used similarly in a study conducted by Maheshwari et.al to assess the psychological impact in COVID-19 survivors [11].

Corresponding to the time of discharge from the hospital, varying levels of anxiety were found among the subjects, with a majority of them having mild anxiety, followed by severe anxiety and mild to moderate anxiety. The major contributors to the severe as well as mild to moderate anxiety were impaired intellectual abilities, insomnia, and depressed mood. It was also noted that females represented a higher percentage across all categories of anxiety. The subsequent trends showed a notable improvement in anxiety scores at each year, with the mean score falling drastically at each year, suggesting a positive trend in mental health recovery. However, mild anxiety, mostly owing to disturbed mood and insomnia, continued to persist even in the last year (2024). The present study also showed no statistically significant influence of age or gender on the total HAM-A scores, hence warranting further investigation into sociodemographic differences in post-COVID mental health outcomes.

The findings of the present study were consistent with previous studies done by Kibria et al. and Gramaglia et al. showed the prevalence of anxiety ranging from 20% to 34% among COVID-19 survivors [12,13]. The initial high levels of anxiety observed in this study were consistent with the findings of Clemente-Suárez et al. and Moreno et al., indicating heightened psychological distress during the early recovery periods [14,15]. In the study by Burkauskas et al., anxiety was found to be a common and increasing symptom in post-COVID-19 syndrome, influenced by illness severity, prior psychiatric history, and psychosocial factors [16]. It was recommended to be addressed through targeted assessment and treatment. At one-year follow-up, Mazza et al. also reported pathological fatigue among 33% of COVID-19 survivors [17]. The study by Badinlou et al. reported that anxiety and insomnia that developed with COVID-19 remained stable over time, without any significant improvement [18]. In another longitudinal study, Schäfer et al. found that more than 55% of post-COVID survivors showed clinically relevant symptoms of depression, nearly three-fourths had persistent somatic symptoms, one-fifth continued to show anxiety symptoms, and about 9 in 10 had fatigue at 17 months and 22 months follow-up [19]. Their study identified old age and previous psychiatric illness as the risk factors for severe depression. Guillen-Burgos et al. conducted a similar prospective study investigating the mental health outcomes in COVID-19 survivors in a 24-month follow-up [20]. They reported persistence of anxiety in 16%, depression in 22%, post-traumatic stress disorder (PTSD) in 35%, and insomnia in 24% of them. Similarly, Cacciatore et al. studied post-COVID disability using the World Health Organization Disability Assessment Schedule (WHODAS-12) and reported that more than 28% of the WHODAS-12 variation was due to cognitive dysfunction, anxiety, fatigue, and hyposmia [21].

Overall, the strength of the study lies in its longitudinal design, conducted in an ambispective manner. This enabled us to assess the mental health sequelae, like anxiety, over a period of three years (2021-24). The use of a standardized questionnaire (HAM-A score) further enhances the reliability of the assessments. In addition to this, the exclusion criteria ensured that preexisting psychological or chronic health conditions could not confound the results. This study also addresses the research gap, which shows that most of the studies assessed the mental health sequelae only until one year after developing COVID-19 [22]. 

The study has certain limitations. As the study was conducted in a single center, this could have limited the generalizability. In addition to this, as a part of the data was assessed retrospectively, it might have introduced a possibility of recall bias in the study. Moreover, about 17.4% (19) of the 109 participants showed loss to follow-up. This reduced the final sample size to 90, which was close to the calculated sample size of 92 for this study. While known confounders were controlled using the restriction method by excluding individuals with preexisting psychological issues and chronic health conditions from the study, unknown confounders like socioeconomic disparities (as the patients are Employees' State Insurance Corporation Plan-insured person (ESIC-IP) card holders) among the patients could not be controlled. These factors may have influenced the outcomes of the study. Future studies can include a control group to address the confounders effectively. Lastly, the observed trends in the HAM-A scores of the participants can be attributed to various factors. So, future studies can consider having a biopsychosocial framework. 

## Conclusions

The present study traced the trajectory of anxiety symptoms over time, demonstrating significant improvement in the mental health status of the COVID-19 survivors, as measured by their HAM-A scores. This highlights the importance of monitoring and addressing anxiety symptoms in COVID-19 survivors. The study findings will be beneficial in the development of public health strategies, such as digital mental health platforms and primary care mental health screening. 

## Appendices

Appendix 1

**Table 5:** Questionnaire for prevalence of anxiety amongst COVID-19 survivors Rating scale: 0: Not present; 1: Mild; 2: Moderate; 3: Severe; 4: Very severe

Section/Question Type	Item	Response (0-4 Scale)
Consent	Consent	Yes/No
Name	Name	
Demographics	Age	
	Sex	
Date	Date	
Anxiety Symptoms	1) ANXIOUS MOOD - worries, anticipation of the worst, fearful anticipation, irritability	
	2) TENSION - feelings of tension, fatigability, startle response, moved to tears easily, trembling, feelings of restlessness, inability to relax	
	3) FEARS - of dark, of strangers, of being left alone, of animals, of traffic, of crowds	
	4) INSOMNIA - Difficulty in falling asleep, broken sleep, unsatisfying sleep, fatigue on waking, dreams, nightmares, night terrors	
	5) INTELLECTUAL - difficulty in concentration, poor memory	
	6) DEPRESSED MOOD - loss of interest, lack of pleasure in hobbies, depression, early waking, diurnal swing	
	7) SOMATIC (MUSCULAR) - Pains and aches, twitching, stiffness, myoclonic jerks, grinding of teeth, unsteady voice, increased tone	
	8) SOMATIC (SENSORY) - Tinnitus, blurring of vision, hot and cold flushes, feeling of weakness, pricking sensation	
	9) CARDIOVASCULAR SYMPTOMS - tachycardia, palpitations, pain in the chest, throbbing of vessels, fainting feelings, missed beat	
	10) RESPIRATORY SYMPTOMS - pressure or constriction in chest, choking feelings, sighing, dyspnea	
	11) GASTROINTESTINAL SYMPTOMS - dysphagia, abdominal pain, burning sensations, abdominal fullness, nausea, vomiting, borborygmi, loose stools, loss of weight, constipation	
	12) GENITOURINARY SYMPTOMS - frequency of micturition, urgency of micturition, amenorrhea, menorrhagia, frigidity, premature ejaculation, loss of libido, impotence	
	13) AUTONOMIC SYMPTOMS - dry mouth, flushing, pallor, tendency to sweat, giddiness, tension, headache, raising of hair	
	14) BEHAVIOR AT INTERVIEW - fidgeting, restlessness or pacing, tremors of hands, furrowed brow, strained face, sighing or rapid respiration, facial pallor, swallowing	
